# Circular RNA circ-BNC2 (hsa_circ_0008732) inhibits the progression of ovarian cancer through microRNA-223-3p/ FBXW7 axis

**DOI:** 10.1186/s13048-022-01025-w

**Published:** 2022-08-14

**Authors:** Ting Liu, Li Yuan, Xiaofeng Zou

**Affiliations:** Department of Gynaecology, Affiliated Hospital of Zunyi Medical University, Dalian Road No.149, Huichuan District, 563000 Zunyi, Guizhou Province China

**Keywords:** Ovarian cancer, circ-BNC2, miR-223-3p, FBXW7, Proliferation

## Abstract

**Background:**

Circular RNAs (circRNAs) are reported to be key regulators in the progression of human cancers. This work focuses on the function and molecular mechanism of circRNA-BNC2 (circ-BNC2) (also known as hsa_circ_0008732) in ovarian cancer (OC).

**Methods:**

Quantitative real-time polymerase chain reaction (qRT-PCR) was conducted to detect circ-BNC2, microRNA-223-3p (miR-223-3p) and F-box and WD repeat domain containing 7 (FBXW7) mRNA expressions in OC tissues and cells. Besides, cell counting kit 8 (CCK-8), transwell assay and cell cycle assays were executed to assess the proliferative, migrative, invasive abilities, and cell cycle progression of OC cells, respectively. Dual-luciferase reporter gene assay and RNA pull-down assay were used to validate the targeting relationships between miR-223-3p and circ-BNC2 or FBXW7. Western blot was adopted to determine FBXW7 protein levels in OC cells.

**Results:**

Circ-BNC2 expression was downregulated in OC tissues and cell lines, which was associated with higher FIGO stage and lymph node metastasis of OC patients. Circ-BNC2 overexpression repressed the proliferation, migration, invasion of OC cells and induced cell cycle arrest, while silencing circ-BNC2 worked oppositely. Mechanistically, circ-BNC2 could upregulate FBXW7 expression in OC cells via sponging miR-223-3p.

**Conclusion:**

Circ-BNC2 suppresses the progression of OC via regulating miR-223-3p / FBXW7 axis. Our findings provided potential biomarker for OC therapy.

## Introduction

Ovarian cancer (OC) is a common gynecological malignant disease, with 5-year survival rate of 44% [1]. In United States, approximately 21,750 women are diagnosed with OC and 13,940 women die of OC each year [2]. Patients with OC have atypical early symptoms, making early diagnosis and treatment difficult [3, 4]. At present, the combination of surgery and chemotherapy is commonly applied in clinical treatment of OC, instead, the prognosis of most patients still is pessimistic [5]. Therefore, it is important to study the underlying molecular mechanism of OC carcinogenesis so as to improve the diagnosis and treatment.

Circular RNAs (circRNAs) are a category of endogenous non-coding RNA transcripts with covalently closed loop structures, which can be composed of exons, introns or intergenic regions of protein-coding genes [6]. Due to the lack of 3’ poly (A) tail and 5’ end cap, circRNAs are resistance to RNase R and are highly stable in vivo compared with linear RNA transcript [7]. Many studies have reported that circRNAs can serve as molecular sponge of microRNAs (miRNAs) to modulate gene expressions in the progression of cancers [8–12]. For example, circ-WHSC1 promotes the development of endometrial cancer by targeting miR-646 and up-regulating the expression of NPM1 [10]. In addition, circRNA-BNC2 (circ-BNC2 or hsa_circ_0008732) has been reported to be down-regulated in the plasma of patients with epithelial OC [13]. However, the biological function and underlying mechanism of circ-BNC2 in OC has not been clarified yet.

MiRNAs are endogenous non-coding RNAs, approximately 20–25 *nt* [14]. MiRNAs modulate gene expression at post-transcriptional level via repressing messenger RNA (mRNA) translation or inducing mRNA degradation [15]; the dysregulation of miRNAs is involved in the pathogenesis of multiple human diseases such as cardiovascular diseases, metabolic diseases, and cancers [15]. It is reported that, miRNA-223-3p (miR-223-3p) often serves as a cancer-promoter [16–18]. For example, miR-223-3p facilitates the malignant biological behaviors of colon cancer cells via negatively modulating PR/SET domain 1 [17]. MiR-223-3p promotes the proliferation and invasion of OC cells via targeting SRY-box transcription factor 11 [19]. This work is aimed to investigate the biological function and underlying molecular mechanism of circ-BNC2 in OC. We report that circ-BNC2 represses the development of OC by sponging miR-223-3p and upregulating F-box and WD repeat domain containing 7 (FBXW7) expression.

## Materials and methods

### Ethics statement and patient samples

40 pairs of OC tissues and adjacent tissue samples were collected from patients who were diagnosed with OC and received surgical resection in the Affiliated Hospital of Zunyi Medical University. None of the patients received any anti-cancer treatment before surgery, and all samples were immediately frozen in liquid nitrogen and stored at -80℃. This study, with written informed consent, was supported by the Ethics Committee of Affiliated Hospital of Zunyi Medical University.

### Cell culture and transfection

Humans OC cell lines (SKOV3, CAOV-3, OVCAR-3, OV90, HO-8910, ES-2) and normal ovarian epithelial cell line IOSE-80 were available from Shanghai Institute of Cytology, Chinese Academy of Sciences (Shanghai, China) and American Type Culture Collection (ATCC, Rockville, MD, USA). These cells were cultured in Dulbecco’s modified Eagle’s medium (DMEM; Hyclone, Logan, UT, USA) with 10% fetal bovine serum (Gibco, Gran Island, NY, USA), 100 U/mL penicillin and 100 µg/ml streptomycin (Invitrogen, Carlsbad, CA, USA) at 37 °C in 5% CO_2_. Circ-BNC2 overexpression plasmid (oe-circ), pcDNA empty vector (Vector), small interfering RNA (siRNA) targeting circ-BNC2 (si-circ#1 and si-circ#2), siRNA negative control siRNA (si-NC), miR-223-3p mimic and negative control (miR-control) were from Invitrogen. The cell transfection was carried out with Lipofectamine® 3000 according to the manufacture’s protocol.

### RNA extraction and quantitative real-time polymerase chain reaction (qRT-PCR)

Total RNA was extracted from tissues and cells using TRIzol reagent (Yeasen Biotech, Shanghai, China). Reverse transcription of miRNA and mRNA were conducted with the Bulge-Loop™ miRNA qRT-PCR kit (RiboBio, Guangzhou, China) and PrimeScript RT Master Mix kit (TaKaRa, Dalian, China), respectively. Besides, qRT-PCR was performed using a SYBR® Premix Ex Taq™ II kit (TaKaRa, Dalian, China) on an Applied Biosystems Prism 7500 Fast Sequence Detection System (Applied Biosystems, Foster City, CA, USA). Glyceraldehyde 3-phosphate dehydrogenase (GAPDH) and U6 were used as the internal references. The relative expression was calculated by 2 ^− ΔΔCt^ method. The primers are listed in Table 1.

**Table 1:** Primer sequences

Name	Primer sequences
Circ-BNC2	Forward: 5′-GACATGGCAAAACGCTGATA-3′
	Reverse: 5′-TGGCCAGTCTTGCTCACTAA-3′
MiR-223-3p	Forward: 5’-GAAGCTGTACCTAACATACCGTG-3’
	Reverse: 5′-GATTGGTCGTGGACGTGTCG-3′
FBXW7	Forward: 5′-ACTGGAAAGTGACTCTGGGA-3′
	Reverse: 5′-TACTGGGGCTAGGCAACAA-3′
GAPDH	Forward: 5’-TGGTATC GTGGAAGG ACTC-3’
	Reverse: 5’-AGTAGAGGCAGGGATGATG-3’
U6	Forward: 5’-GCACCTTAGGCTGAACA-3’
	Reverse: 5’-AGCTTATGCCGAGCTCTTGT-3’

### Cell counting kit-8 (CCK-8) assay

CCK-8 kit (Beyotime, Shanghai, China) was utilized to detect the proliferative ability of OC cell lines. 48 h after transfection, the trasfected cells were seeded in 96-well plates at the density of 2 × 10^3^ cells / well. 10 µL of CCK-8 solution was added into each well at the indicated time (24th h, 48th h, 72nd h and 96th h) and then the cells were incubated for 2 h at 37 °C. Finally, the absorbance at 450 nm wavelength was examined using a microplate reader (Bio-Rad, Hercules, CA, USA).

### Transwell assay

The transfected OC cells in the logarithmic growth phase were resuspended with serum-free DMEM, with the density adjusted to 3 × 10^5^ cells/mL. 200 µL of cell suspension was subsequently transferred in the upper chamber of a transwell insert (24-well insert; 8 μm pore size; Corning, NY, USA). 600 µL of DMEM containing 10% FBS was loaded into the lower compartment, and the cells were cultured in 5% CO_2_ at 37 °C for 24 h. Next, the cells on the upper surface of the filter were gently wiped off with a cotton swab. Subsequently, the cells on the lower surface were accordingly fixed by 4% paraformaldehyde for 15 min and immediately stained with 0.1% crystal violet solution for 10 min. Then the cells were washed by phosphate buffered solution (PBS). Five high-power fields were randomly selected under a light microscope (Nikon, Tokyo, Japan), and the cells passing through the filter were counted. In the invasion assay, the transwell filter was coated with Matrigel (BD Biosciences, Franklin Lakes, NJ, USA), and the remaining steps were the same as the migration assay.

### Cell cycle assay

48 h after the transfection, OC cells were fixed with 75% ethanol. Next, the cells were immersed in PBS and subsequently incubated with 0.5% Triton X-100 containing 1 mg / mL RNase A at 37 °C for 30 min. Next, the cells were stained with propidium iodide (PI; 50 µg / mL; Sigma-Aldrich, Louis, MO, USA) for 30 min at ambient temperature in darkness. Ultimately, the cell cycle distribution of the cells was analyzed using a FACScan flow cytometer (BD Biosciences, Franklin Lakes, NJ, USA).

### Subcellular localization

Cytoplasmic and nuclear fraction of SKOV3 and HO-8910 cells was isolated using a PARIS™ Kit (Invitrogen, Carlsbad, CA, USA). Briefly, the cells were lysed with cell fractionation buffer and centrifuged at 500 ×g at 4 ℃ for 5 min to separate the nuclear and cytoplasmic cell fractions. The supernatant was transferred to a RNase-free tube. The remaining lysate was washed with cell fractionation buffer and centrifuged again. Then the RNA of cytoplasm and nuclear was eluted with the elution solution. Subsequently, circ-BNC2 expression in the cytoplasm and nucleus of SKOV3 and HO-8910 cells was detected by qRT-PCR, with GAPDH and U6 as cytoplasmic and nuclear controls, respectively.

### Dual-luciferase reporter gene assay

The wild type (WT) and mutant (MUT) sequences of circ-BNC2 or FBXW7 3′-UTR containing putative miR-223-3p binding sites were accordingly sub-cloned into the pGL3-Basic luciferase vectors (Promega, Madison, WI, USA) to construct recombinant reporter plasmids. The above luciferase reporter plasmids were co-transfected with miR-control or miR-223-3p mimic into SKOV3 or HO-8910 cells, respectively. 48 h later, the relative luciferase activity was tested by the Dual-Luciferase Reporter Assay System (Promega, Madison, WI, USA).

### RNA pull-down assay

The biotinylated miR-223-3p was labeled using the RNA 3’ End Desthiobiotinylation Kit (Thermo Fisher Scientific, Waltham, MA, USA). The Pierce Magnetic RNA–Protein Pull-Down Kit (Thermo Scientific, Waltham, MA, USA) was used to perform RNA pull-down assay. Briefly, SKOV3 or HO-8910 cells were lysed with lysis buffer. The biotin-miR-223-3p, or biotin-negative control (biotin-miR-control) were then incubated with cell lysates, followed by the incubation of M-280 streptavidin magnetic beads (Sigma-Aldrich, Louis, MO, USA). Then, the precipitated RNA on the beads was eluted, and subsequently qRT-PCR was performed.

### Western blot assay

Total protein of the transfected OC cells was extracted using radio-immunoprecipitation assay (RIPA) lysis buffer (Beyotime, Shanghai, China), and the concentration of protein was evaluated by a bicinchoninic acid (BCA) protein assay kit (Beyotime, Shanghai, China). After the protein samples were mixed with loading buffer and denatured, the proteins were separated by sodium dodecyl sulfate-polyacrylamide gel electrophoresis (SDS-PAGE) and electrotransfered to polyvinylidene fluoride (PVDF) membranes (Millipore, Billerica, MA, USA). The membranes were subsequently blocked with 5% skimmed milk for 2 h at ambient temperature and incubated with specific primary antibodies overnight at 4 °C. Next day, the membranes were washed and incubated with horseradish peroxidase (HRP)-conjugated goat anti-rabbit IgG H&L (1:5000, ab205718, Abcam, Shanghai, China) for 1 h at ambient temperature. The protein bands were visualized by an enhanced chemiluminescence kit (Promega, Madison, WI, USA) and analyzed by Image-Pro Plus software. GAPDH was regarded as an internal reference. The primary antibodies were: anti-FBXW7 antibody (1:1000, ab109617, Abcam, Shanghai, China), anti-GAPDH antibody (1:2000, ab9485, Abcam).

#### In-vivo model of lung metastasis

The animal experiments were approved by the Animal Care and Use Committee of the Affiliated Hospital of Zunyi Medical University. Six-week-old female BALB/c nude mice (*n* = 24) were purchased from the Laboratory Animal Center of Guizhou Medical University (Guizhou, China) and maintained in a pathology-free environment. For *in-vivo* lung metastasis experiments, SKOV3 cells (1 × 10^6^ cells per mouse) without or with circ-BNC2 overexpression or the control cells were injected into the tail vein of the nude mice (12 mice per group). 21 days after injection, the mice were sacrificed, and the lung tissue was harvested. The lung tissues were fixed, formalin, paraffin-embedded, and sectioned before hematoxylin-eosin (H&E) staining.

### Statistical analysis

All experiments were repeated for three times, with data presented as mean ± standard deviation (SD). Notably, the statistical analyses were accomplished using SPSS 19.0 (IBM, SPSS, Chicago, IL, USA). Independent sample *t*-test was executed for comparisons between two groups, and one-way analysis of variance with post-hoc test was conducted for comparisons among multiple groups. Besides, the overall survival analysis was conducted with Kaplan-Meier plots and log-rank tests. The correlation of gene expression was analyzed with Pearson’s correlation coefficient. Statistically, *P* < 0.05 is significant.

## Results

### Circ-BNC2 is downregulated in OC and correlates with poor prognosis

**Fig. 1:**
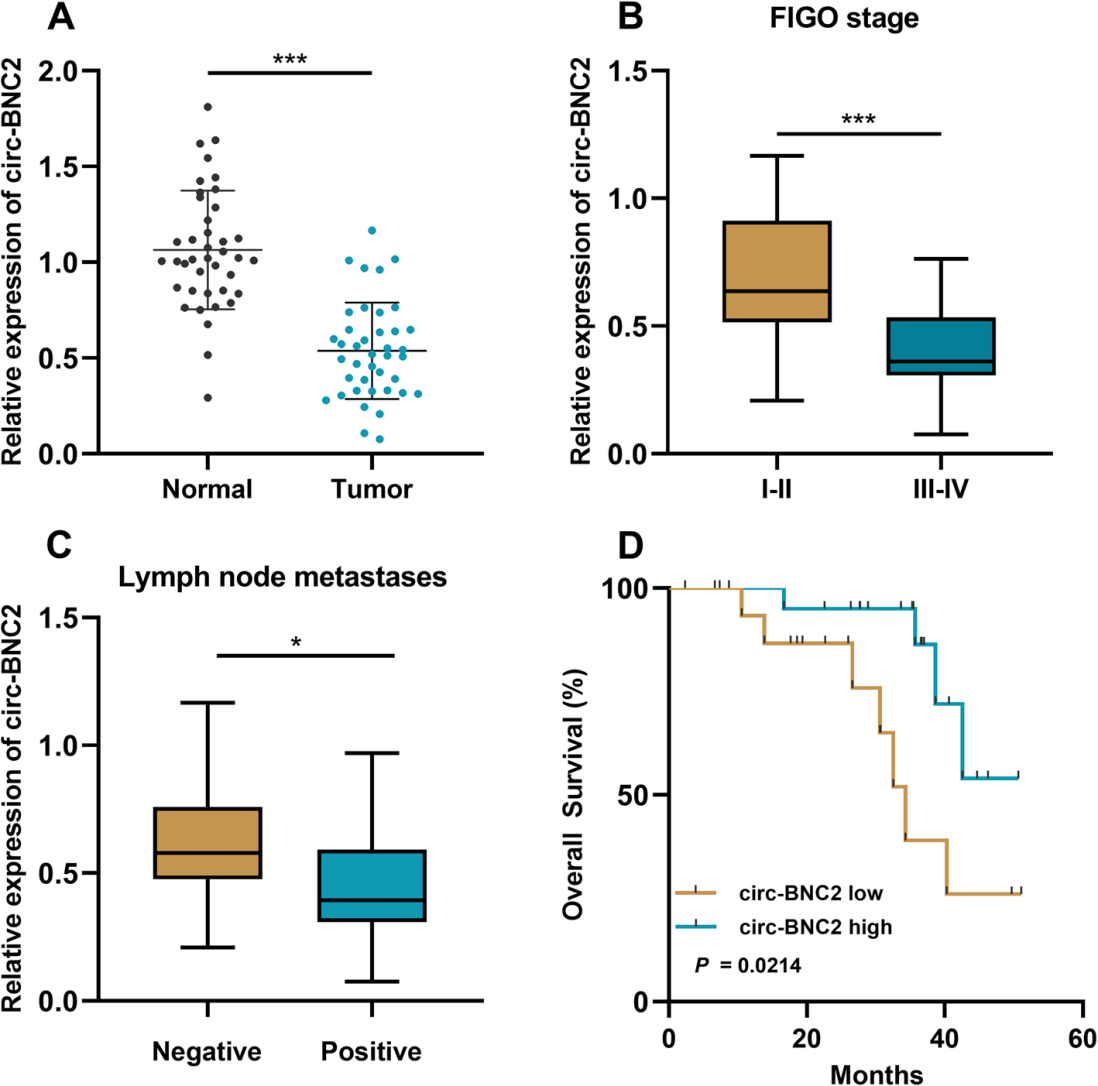
Circ-BNC2 is down-regulated in OC. **A** qRT-PCR was used to detect the expression of circ-BNC2 in 40 pairs of OC tissues and normal tissues adjacent to tumors. **B**-**C** The association between circ-BNC2 expression and FIGO stage (**B**), lymph node metastasis (**C**) in OC tissues. D. Kaplan-Meier curves were used to analyze the correlation between circ-BNC2 expression and the overall survival of OC patients. *P < 0.05, and **P < 0.001

As qRT-PCR showed, circ-BNC2 expression in OC tissues was markedly lower than that in the normal tissues adjacent to tumors (Fig. 1A). In OC tissues, the low expression of circ-BNC2 was associated with the advanced Federation International of Gynecology and Obstetr (FIGO) stage and positive lymph node metastasis of the patients (Fig. 1B and C). Kaplan-Meier curve analysis indicated that low circ-BNC2 expression was associated with a shorter overall survival time of the OC patients (Fig. 1D).

### Circ-BNC2 inhibits OC cell growth, migration, invasion, and cell cycle progression

**Fig. 2:**
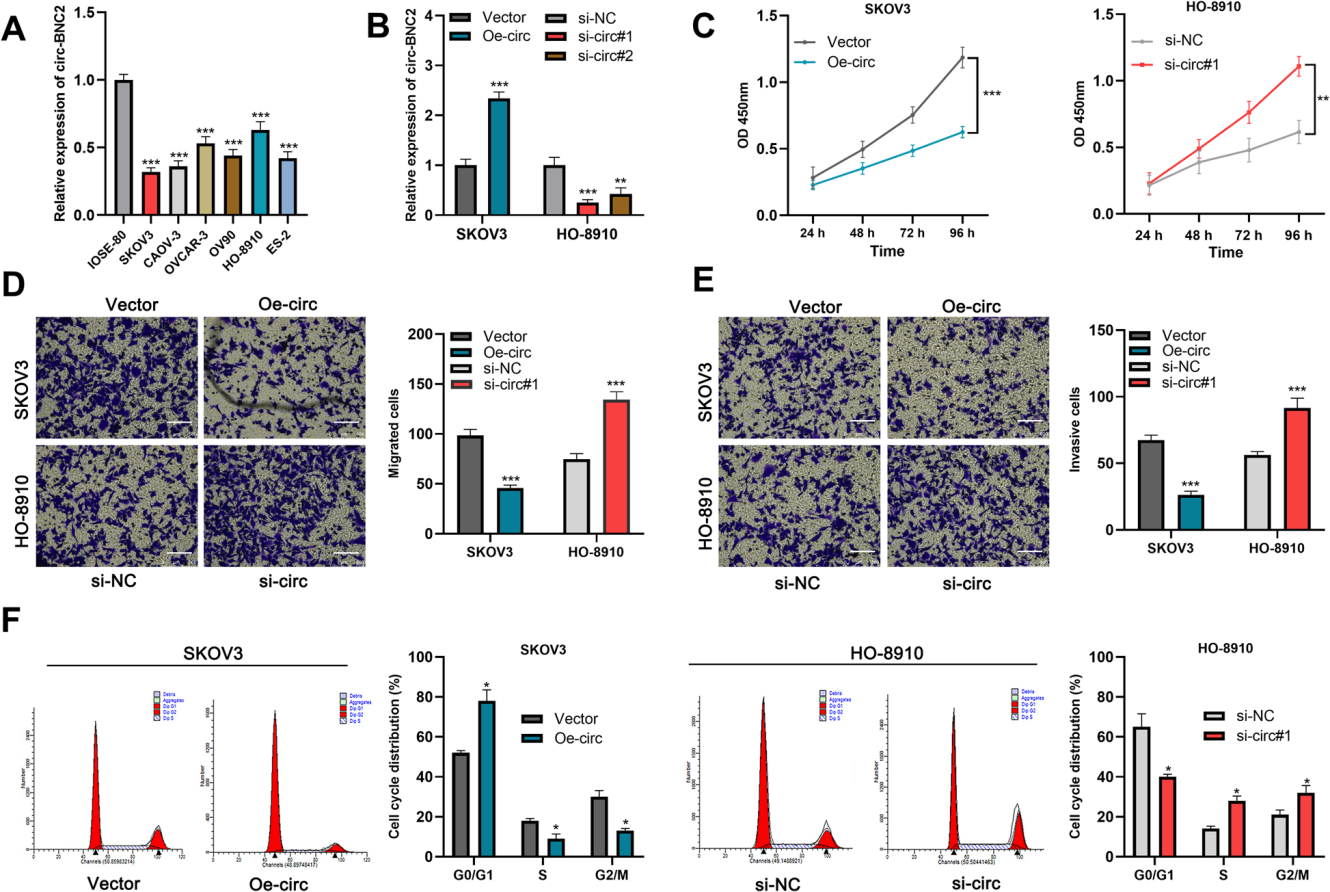
The effects of circ-BNC2 on the proliferation, migration, invasion and cell cycle of OC cells*. ***A** The expression of circ-BNC2 in OC cells (SKOV3, CAOV-3, OVCAR-3, OV90, HO-8910, and ES-2) and normal ovarian epithelial cells IOSE-80 were detected by qRT-PCR. **B** Circ-BNC2 overexpression plasmids were transfected into SKOV3 cells, and circ-BNC2 siRNAs (si-circ#1 and si-circ#2) were transfected into HO-8910 cells, and the expression of circ-BNC2 was detected by qRT-PCR. C. CCK-8 assay was used to detect the proliferative ability of OC cells after transfection. **D** and **E** Transwell assay was used for detecting the migration and invasion of OC cells after transfection. Scale bar: 500 μm. **F** Cell cycle assays was used to detect the cell cycle distribution in OC cells after transfection. * *P* < 0.05, ** *P* < 0.01, and *** *P* < 0.001

Additionally, circ-BNC2 expression was markedly down-regulated in OC cell lines (SKOV3, CAOV-3, OVCAR-3, OV90, HO-8910, and ES-2) as against that of immortalized ovarian epithelial cell line IOSE-80 (Fig. 2A). Next, SKOV3 cells, with lowest circ-BNC2 expression level, and HO-8910 cells, with the highest circ-BNC2 expression level, were selected for subsequent experiments. qRT-PCR showed that circ-BNC2 expression was demonstrably up-regulated in SKOV3 cells transfected with circ-BNC2 overexpression plasmid; and the transfection of si-circ#1 and si-circ#2 significantly decreased circ-BNC2 expression in HO-8910 cells (Fig. 2B). Since si-circ#1 had a higher knockdown efficiency, so it was selected for subsequent experiments. CCK-8 assay showed that circ-BNC2 overexpression contributed to a great decrease in the proliferative ability of SKOV3 cells; in contrast, knockdown of circ-BNC2 significantly promoted the proliferation of HO-8910 cell (Fig. 2C). Transwell assay showed that circ-BNC2 overexpression led to reduced migration and invasion of SKOV3 cells; the depletion of circ-BNC2 significantly enhanced the migrative and invasive abilities of HO-8910 cells (Fig. 2D and E). In addition, cell cycle assays showed that circ-BNC2 overexpression resulted in cell cycle arrest; while knockdown of circ-BNC2 accelerated the transition of HO-8910 cells from G0/G1 phase into S and G2/M phases (Fig. 2F). Collectively, these data suggest that circ-BNC2 inhibits the growth, migration, and invasion of OC cell, and induces G0/G1 phase arrest.

### Circ-BNC2 adsorbs miR-223-3p

**Fig. 3:**
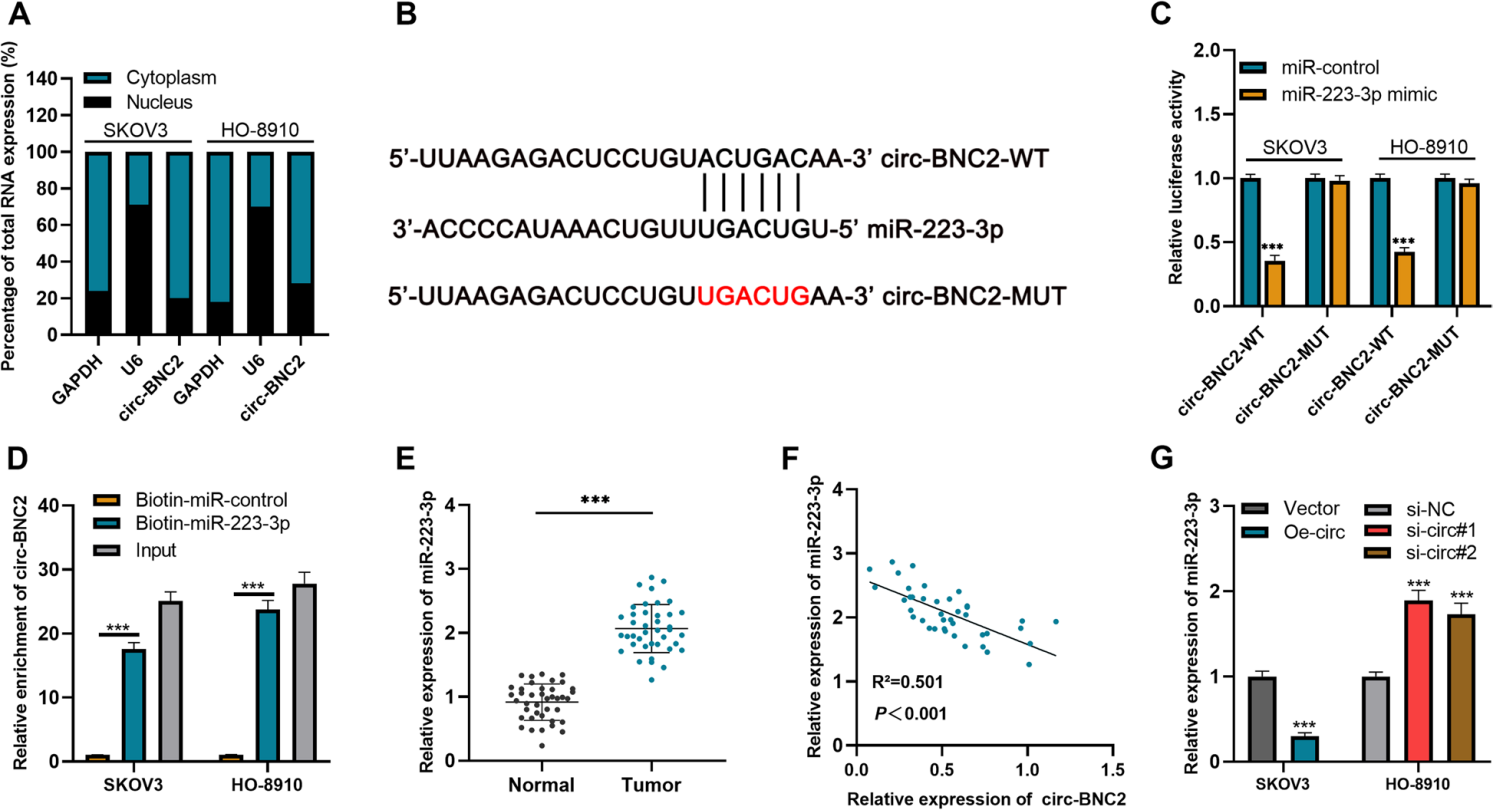
Circ-BNC2 acts as a sponge of miR-223-3p*. ***A** The subcellular fractionation assay was used to detect the expression of circ-BNC2 in the nucleus and cytoplasm of SKOV3 and HO-8910 cells. **B** Bioinformatics tools was used to predict the binding site of circ-BNC2 and miR-223-3p. **C** Dual-luciferase reporter gene assay was used to validate the specific binding between circ-BNC2 and miR-223-3p in SKOV3 and HO-8910 cells. **D** RNA pull-down assay was carried out to verify the interaction between miR-223-3p and circ-BNC2 in SKOV3 and HO-8910 cells. **E** qRT-PCR was used to detect the expression of miR-223-3p in OC tissues and normal tissues adjacent to tumors. *n* = 40. **F** Pearson’s correlation coefficient analysis was performed to analyze the correlation between circ-BNC2 and miR-223-3p expression in OC tissues. **G** qRT-PCR was used to detect the expression of miR-223-3p in OC cells after silencing or overexpressing circ-BNC2. ***P < 0.001

CircRNAs frequently function as molecular sponge for miRNAs [11]. Circ-BNC2 was mainly distributed in the cytoplasm of SKOV3 and HO-8910 cells (Fig. 3A), suggesting that it could probably function as a competitive endogenous RNA. Circinteractome database (https://circinteractome.nia.nih.gov/) was used to predict the potential target miRNAs of circ-BNC2. There was a complementary sequence between miR-223-3p and circ-BNC2 (Fig. 3B). Dual-luciferase reporter gene assay showed that miR-223-3p could dramatically suppressed the relative luciferase activity of circ-BNC2-WT group, but had no significant effect on that of circ-BNC2-MUT group (Fig. 3C). RNA Pull-down assay suggested that circ-BNC2 could be pulled down by biotin-miR-223-3p (Fig. 3D). qRT-PCR showed that miR-223-3p expression in OC tissue was higher than that in the normal tissues adjacent to tumor (Fig. 3E). Additionally, there was a negative correlation between miR-223-3p and circ-BNC2 expressions in OC tissues (Fig. 3F). Additionally, miR-223-3p expression was inhibited in SKOV3 cells transfected with circ-BNC2 overexpression plasmid; however, miR-223-3p expression was dramatically increased in HO-8910 cells with circ-BNC2 knockdown (Fig. 3G). Collectively, it is concluded that miR-223-3p is the direct target gene of circ-BNC2.

### Circ-BNC2 inhibits the progression of OC cells by sponging miR-223-3p

**Fig. 4:**
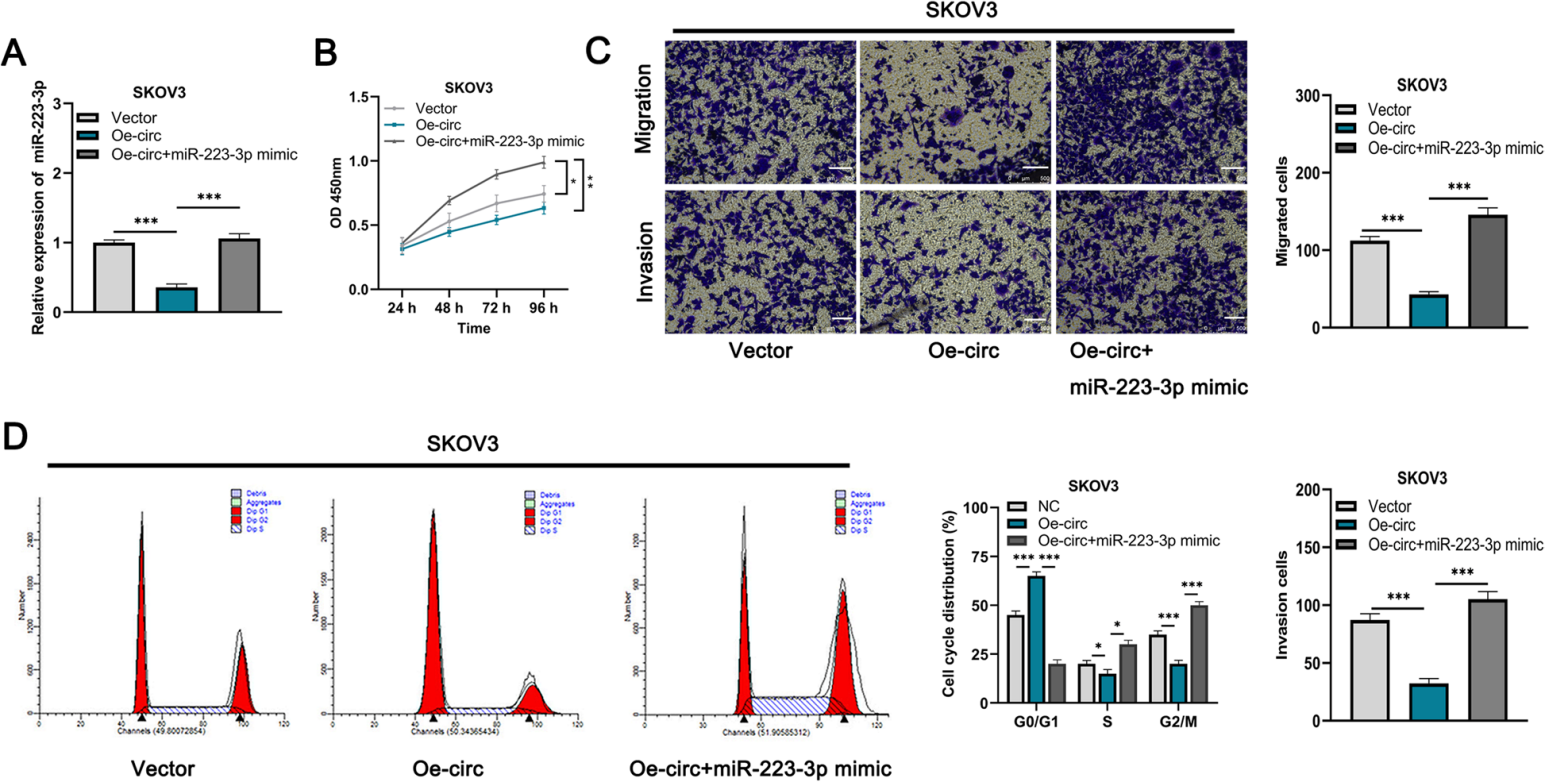
Circ-BNC2 inhibits the progression of OC cells by sponging miR-223-3p. **A** SKOV3 were transfected with negative control (NC), circ-BNC2 overexpression plasmid (Oe-circ), Oe-circ + miR-223-3p mimics, and qRT-PCR was applied to detect the expression of miR-223-3p in SKOV3 cells after transfection. **B** CCK-8 assay used to detect the proliferation of SKOV3 cells after transfection. **C** Transwell assay was used to detect the migration and invasion of SKOV3 cells after transfection. Scale bar: 500 μm. **D** Cell cycle assays used to detect the cell cycle distribution of SKOV3 cells after transfection. *P < 0.05, **P < 0.01, and ***P < 0.001

To further clarify the mechanism of circ-BNC2 and miR-223-3p in regulating the proliferation, migration, invasion and cell cycle of OC cell, functional compensation experiments were performed. MiR-223-3p expression was increased after transfection of miR-223-3p mimics into SKOV3 cells with circ-BNC2 overexpression (Fig. 4A). CCK-8 and Transwell assays showed that the growth, migration and invasion of SKOV3 cell were significantly reduced after circ-BNC2 overexpression, while transfection with miR-223-3p mimics counteracted this effect (Fig. 4B and C). Cell cycle assay showed that after transfection of miR-223-3p mimic into SKOV3 cells with circ-BNC2 overexpression, the number of cells in G0 / G1 phase decreased, while that of cells in S phase and G2 / M phase was increased (Fig. 4D). The above results indicate that circ-BNC2 inhibits the progression of OC depending on miR-223-3p.

### Circ-BNC2 up-regulates the expression of FBXW7 via absorbing miR-223-3p

**Fig. 5:**
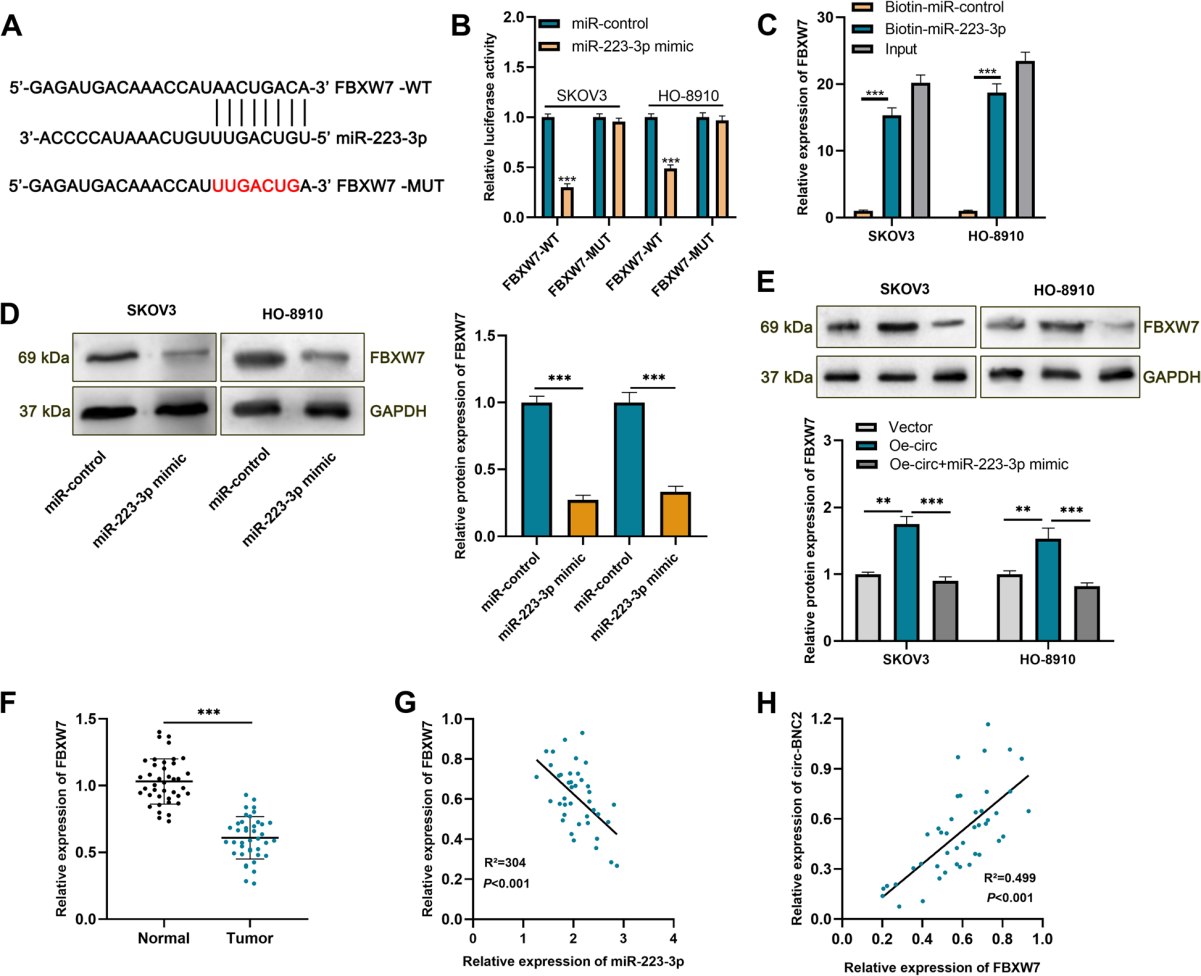
Circ-BNC2 increases the expression of FBXW7 by targeting miR-223-3p*. ***A** TargetScan online tool was used to predict the binding site between miR-223-3p and FBXW7 3′-UTR. **B** Dual-luciferase reporter gene assay was used to validate the targeting relationship between miR-223-3p and FBXW7 3′-UTR. **C** RNA pull-down assay was carried out to verify the interaction between miR-223-3p and FBXW7 in SKOV3 and HO-8910 cells. **D**-**E** Western blot assay was used to examine the protein expressios of FBXW7 in SKOV3 and HO-8910 cells after transfection. **F** qRT-PCR was used to detect the expression of FBXW7 mRNA in OC tissues and normal tissues adjacent to tumors. *n* = 40. **G**-**H** Pearson’s correlation coefficient was used to analyze the correlation between miR-223-3p and FBXW7 mRNA, FBXW7 mRNA and circ-BNC2 expression in OC tissues, respectively. **P < 0.01, and ***P < 0.001

To further study the mechanism of circ-BNC2/miR-223-3p axis in the progression of OC, we used TargetScan database to predicted target genes of miR-223-3p, and FBXW7 was identified as a candidate downstream target of miR-223-3p (Fig. 5A). Besides, dual-luciferase reporter gene assay proved that miR-223-3p mimic decreased the luciferase activity of FBXW7-WT group, but had no significant effect on that of FBXW7-MUT group (Fig. 5B). RNA pull-down assay highlighted that biotin-miR-223-3p could enrich FBXW7 in SKOV3 and HO-8910 cells (Fig. 5C). Western blot indicated that circ-BNC2 overexpression increased the protein expression of FBXW7, and miR-223-3p overexpression repressed the levels of FBXW7 protein in SKOV3 and HO-8910 cells (Fig. 5D-E). Notably, FBXW7 expression level was lower in OC tissues than that of normal tissues adjacent to tumor (Fig. 5F). In addition, there was a negative correlation between miR-223-3p expression and FBXW7 expression in OC tissues (Fig. 5G); FBXW7 expression was positively correlated with the expression of circ-BNC2 (Fig. 5H). Taken together, FBXW7 is a target of miR-223-3p, and circ-BNC2 upregulates FBXW7 expression in OC cells via targeting miR-223-3p.

### Overexpression of circ-BNC2 inhibits the lung metastasis of OC cells in vivo

**Fig. 6:**
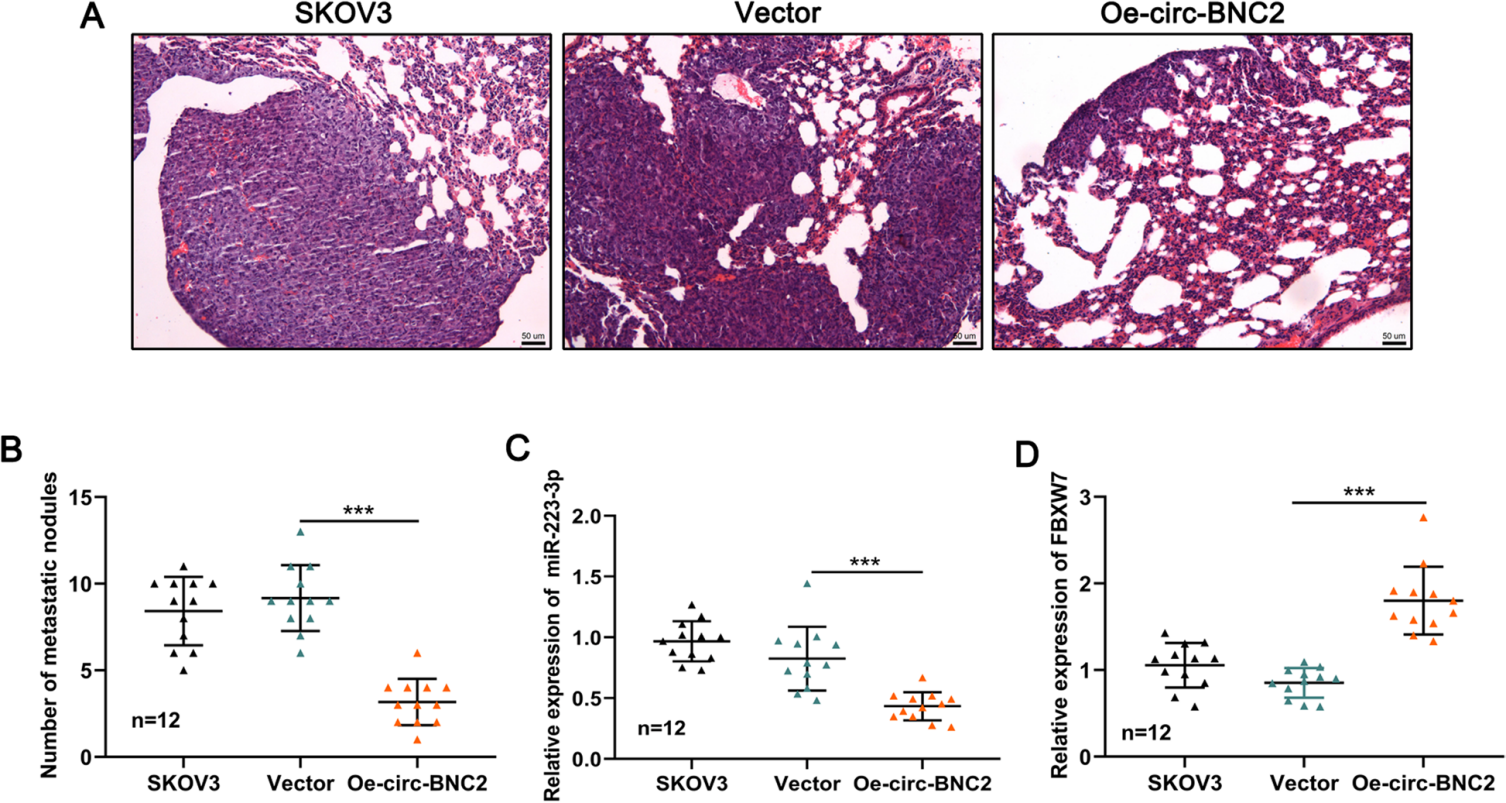
Circ-BNC2 inhibits the lung metastasis of OC cells in vivo. **A**-**B** Metastatic nodules in mice were detected by H&E staining after tail vein injection of circ-BNC2-overexpressing SKOV3 cells. *n* = 12, scale bar: 50 μm. **C**-**D** qRT-PCR was used to detect the expression of miR-223-3p and FBXW7 in the lung tissues of mice in the circ-BNC2 overexpression group and the vector group. **P < 0.01, and ***P < 0.001

To further elucidate the effect of circ-BNC2 on the progression of OC, SKOV3 cells transfected without or with circ-BNC2 overexpression plasmid and control plasmid were injected into the tail vein of nude mice, respectively, to establish a lung metastasis model. The H&E staining of mouse lung tissue showed that metastatic nodules were significantly reduced in the circ-BNC2 overexpression group compared with the vector group (Fig. 6A-B). In addition, compared with the vector group, miR-223-3p expression was down-regulated and FBXW7 expression was up-regulated in the mouse lung tissue of the circ-BNC2 overexpression group (Fig. 6C-D). In short, circ-BNC2 can inhibit OC metastasis in vivo.

## Discussions

CircRNAs are an emerging class of RNA molecules, which play important roles in human diseases [20–25]. It has been reported that circRNAs are important regulators in cancer biology [25]. For example, circ_001783 enhances the proliferative and migrative abilities of breast cancer cells through sponging miR-200c-3p [26]. High circ_006100 expression is closely associated with high tumor node metastasis (TNM) stage, poor cellular differentiation and lymph node metastasis of patients with gastric cancer, and it expedites the growth and metastasis of gastric cancer cells by adsorbing miR-195 [27]. Also, circ_0015756 directly targets miR-942-5p to up-regulate cullin 4B expression and inhibits the apoptosis of OC cells [28]. Here we proved that circ-BNC2 expression was downregulated in OC tissues and cell lines, and patients with a low circ-BNC2 expression had a poor prognosis. In addition, overexpression of circ-BNC2 restrained the viability, migration, invasion, and cell cycle progression of OC cells, while knocking down circ-BNC2 had the opposite effect. In addition, circ-BNC2 overexpression could inhibit the lung metastasis of OC cells in vivo. Collectively, circ-BNC2 is identified as a novel tumor suppressor in the progression of OC.

Reportedly, some circRNAs function as miRNA sponges and can modulate expression of tumor suppressor or promoter through the circRNA-miRNA-mRNA network [29, 30]. In this study, miR-223-3p was confirmed to be a target of circ-BNC2. It is reported that miR-223-3p plays different roles in different human malignancies [31]. For example, in non-small cell lung cancer, miR-223-3p restrains the malignant biological behaviors of tumor cells and induces the apoptosis via directly targeting ras homolog family member B [32]. MiR-223-3p blocks the multiplication and metastasis of oral squamous cell carcinoma cells by inhibiting the expression of short stature homeobox 2 [33]. Conversely, miR-223-3p enhances the proliferative and migrative abilities of renal clear cell carcinoma cells via down-regulating the expression of solute carrier family 4 member 4 [18]. MiR-223-3p promotes the malignant biological behaviors of breast cancer cells through Hippo/Yap signal pathway [34]. In this work, it was revealed that miR-223-3p was highly expressed in OC tissues, which is consistent with previous findings [19]. MiR-223-3p is negatively modulated by circ-BNC2. In addition, miR-223-3p up-regulation could counteracted the inhibitory impact of circ-BNC2 overexpression on the proliferation, migration, invasion and cell cycle of OC cells. This shows that circ-BNC2 can inhibit the progression of OC through sponging miR-223-3p.

Here we found multiple potential targets of miR-223-3p with TargetScan online prediction. Among them, FBXW7 is reported as a tumor-suppressor in OC [35]. We confirmed that miR-223-3p directly targeted and suppressed FBXW7 expression in OC cells. FBXW7 is a component of SKP1-CUL1-F-box protein (SCF) complex, which can mediate the ubiquitination of many oncogenic proteins [36, 37]. FBXW7 regulates cell fate by controlling proteasome-mediated degradation of many oncoproteins (such as c-MYC, NOTsH, KLF5, cyclin E, c-JUN and MCL1) [38]. Reportedly, FBXW7 is often implicated in the progression of diverse cancers as a tumor suppressor. For example, FBXW7 inhibits the epithelial-mesenchymal transformation of oral squamous cell carcinoma cells through PI3K/AKT signaling pathway [39]. FBXW7 can affect the multiplication and apoptosis of colorectal cancer cells through Notch and Akt/mTOR signaling pathways [40]. Another study reports that FBXW7 restrains the invasion and migration of OC cells [41]. The present study proved that FBXW7 expression was declined in OC tissues. It was also revealed that, FBXW7 expression in OC tissues was negatively correlated with miR-223-3p expression, but positively to circ-BNC2 expression. Our data suggest that circ-BNC2 can strengthen FBXW7 expression through absorbing miR-223-3p in OC cells.

## Conclusion

In summary, circ-BNC2 is down-modulated in OC tissues and cell lines. Low circ-BNC2 expression predicts a worse prognosis of OC patients. Mechanistically, circ-BNC2 up-regulates FBXW7 expression by targeting miR-223-3p, thus inhibiting the proliferative, migrative and invasive abilities of OC cell and inducing the cell cycle arrest. This study is helpful to clarify the potential molecular mechanism involved in the progression of OC.

## Data Availability

The data used to support the findings of this study are available from the corresponding author upon request.

## Abbreviations

OC: ovarian cancer
circRNAs: circular RNAs
miRNAs: microRNAs
circ-BNC2: circRNA-BNC2
mRNA: messenger RNA
miR-223-3p: miRNA-223-3p
FBXW7: F-box and WD repeat domain containing 7
ATCC: American Type Culture Collection
DMEM: Dulbecco's modified Eagle's medium
Oe-circ: circ-BNC2 overexpression plasmid
Vector: pcDNA empty vector
siRNA: small interfering RNA
si-circ#1 and #2: siRNA targeting circ-BNC2#1 and #2
si-NC: siRNA negative control siRNA
miR-control: miRNA negative controls
qRT-PCR: quantitative real-time polymerase chain reaction
GAPDH: glyceraldehyde 3-phosphate dehydrogenase
CCK-8: cell counting kit-8
PBS: phosphate buffered solution
PI: propidium iodide
WT: wild type
MUT: mutant
RIPA: radio-immunoprecipitation assay
BCA: bicinchoninic acid
SDS-PAGE: sodium dodecyl sulfate-polyacrylamide gel electrophoresis
PVDF: polyvinylidene fluoride
HRP: horseradish peroxidase
SD: standard deviation
FIGO: Federation International of Gynecology and Obstetr
ceRNA: competitive endogenous RNA
TNM: tumor node metastasis
SCF: SKP1-CUL1-F-box
